# On the Paralysis of the Insane
*"American Journal of Med. Sciences." Editor, Isaac Hays, Philadelphia.


**Published:** 1858-07-01

**Authors:** Pliny Earle


### Art. IV.—ON THE PARALYSIS OF THE INSANE.*

BY PLINY EARLE, M.D.

In previous issues of the *American Journal of the Medical Sciences*, I published two series of cases of that peculiar disease termed by the French physicians, *Paralysie générale*, and by the English and the Americans, *Paralysis of the insane*, but for which I ventured to suggest the name, *Partio-general paralysis.*

Those cases included all the distinctive characteristics of the disease, and the number of autopsies was sufficient to furnish a pretty clear idea of its cerebral pathology. Most of the cases, a Report of which it is proposed to include in this article, present peculiarities, or exceptional characteristics, which render them worthy of preservation. In the one first presented, the disease ran its course so rapidly that the patient was exhausted before the extreme symptoms of paralysis had appeared.

* "American Journal of Med. Sciences." Editor, Isaac Hays, Philadelphia.

Case I.—Mr. —— was born in the interior of the State of New York. He was of medium stature; his hair was light-brown, his eyes blue, and his temperament bilious-nervous.

He learned the business of printing, and worked at it for some years. His intellect was above mediocrity, and his acquirements became such, that at length, and during a term of several years, he was an assistant editor of a newspaper in the city of New York.

In business, he was industrious and persevering; in habits, generally esteemed correct, although, from early life, it is supposed that he gave a pretty free rein to the venereal propensity. He also drank wine, but perhaps never to intoxication. He was married, and had several children. It is said that his parents were both "eccentric," if not insane.

In the summer of 1847, it was observed that he had become unnaturally irritable. This disposition increased upon him through the ensuing autumn and winter, and in the spring there were some evident symptoms of insanity. He, however, continued in his business until about the 1st of May, when the disease prevented the further performance of his duties. On the 10th of May, at the age of forty-two years, he was brought to the Bloomingdale Asylum.

*Condition on Admission.*—He is restless, excited, and incessantly talking, if any one be present. Countenance animated; pupils contracted, unequal—that of the right eye the smallest; tongue moist, pallid, smooth, and very slightly coated; pulse considerably accelerated. No abnormal sound of the heart.

*May* 11*th*. He occupies one of the best rooms, and, if alone, is quiet. He says the Common Council will give this Asylum to him. He will have four hundred mechanics here, and will raise vegetables enough to supply the city. He will want two or three clerks, and three secretaries. He will give ten thousand dollars to stay three weeks and carry out his plans; or he will buy the place in less than a week, pay one hundred and fifty thousand dollars for it, which will not be more than a cent to him, will have all luxuries, and supply all the other patients with them, and will cure all the patients by a special course of treatment.

He has a very slight impediment in his speech. In the midst of conversation, he stops to whistle or to sing.

13*th*. He says he is the cream of American patriotism, and that God has revealed to him all the events of the last six weeks. He is restless, loquacious, petulant; sheds tears, and asks if Washington is not here.

14*th*. He mentions the names of several attendants and patients, claims them as his illegitimate brothers, and offers each of them "a carriage, horses, and twenty thousand dollars, to start upon." Says that he shall be the next President of the United States, and that the Supreme came down last night, and rested on the window-sash, and is still in that cloud (pointing upwards through the window), ready to come down at his bidding.

15*th*. He asserted that he is the "Duke of Gloucester, and entitled to the throne of England, of which Victoria is not the legal possessor."

A few minutes afterwards, he said he was President of the United States and King of England; that his legs are iron, and that he wound up the sun yesterday.

16*th*. He calls one of his fellow-patients the Pope, and to several others gives the titles of some of the English nobility.

20*th*. His excitement has gradually increased from the time of admission. Having become very boisterous, both by day and night, and having begun to destroy furniture and clothing, he was now removed to the ward for violent patients.

21*st*. He declares that he is the son of the King of the world; that he was in the Crusades; that the writings of Shakspeare and Scott are merely a record of his life; and that he had a conversation with the Black Prince the night before last.

22*nd*. Says he killed Abel in the garden; that Eve was his mother; that all the people in the world are descended from him; and that the Dutch Queen had such an affection for him, that it made a tumour grow on his right side. He is much excited, very noisy at night, and destroys clothing.

23*rd*. On entering his room, I said: "You are noisy!" "I've a right to be," he answered. "I'm the god of thunder!" His tongue, as usual, is covered with a thin, white, strongly adherent, pasty fur; bowels habitually costive; right pupil smallest—both contracted; pulse 96, regular; sounds of heart, normal; general sensation, obtuse. He has emaciated constantly since admission.

26*th*. He tore his bed to tatters "to find his cattle;" says he can jump over the house, but is so large he cannot go through the door; tells the physician that he can hold him on his little finger, and could sustain the weight of the world if he had a foothold.

Neither his mental nor physical symptoms changed during the early part of June. On the 19th, his scalp, forehead, and right arm, were much tumefied and ecchymosed, as if beaten against the wall. Being asked how it was done, he laughed, and said: "Jesus Christ did it." Towards the end of the month, and in the early part July, he became more emaciated and feeble; his excitement was less constant, but occasionally, even in the latter part of July, he was very turbulent. At the close of the month he was nearly exhausted, all the worst symptoms, both mental and physical, above-mentioned, continuing. Almost the last words he uttered were an assertion that he was one of the men mentioned in the Old Testament. Died, August 2nd, 1848.

*Treatment.*—Purgatives, alteratives, and tonics. A seton was inserted in the back of the neck on the 23rd of May, and continued until his death. The discharge from it was never copious. Regardless of all medication, the disease regularly proceeded towards its fatal termination.

*Autopsy, sixteen hours after death.*—Pericranium pretty strongly attached to the skull, and but little blood in the vessels. Cranium adheres more than normally strong to the dura mater. It is of ordinary thickness, and not unusually hard. The dura mater adheres to the subjacent membranes on the anterior lobes, and for three inches over the vertex, on the border of each hemisphere, beside the longitudinal sinus. The latter attachments can be separated by dissection alone. The whole brain, when removed from its cavity, appears unnaturally soft or flaccid, and its weight, when laid upon its base, partially tears asunder the corpus callosum. The arachnoid is thickened, semi-opaque, and strongly adherent to the pia mater upon the whole surface of the cerebrum, except the base, where it is normal. The pia mater adheres so strongly to the cortical substance, that on removal it brings off small patches of it. Blood-vessels not remarkably injected. The cortical matter is of normal colour, but is decidedly softened. The brain being cut, the surface of the medulla is interspersed with some bloody points, but they are not numerous. The corpora striata, and the medullary matter around them, are thought to be somewhat softened—the most so in the right hemisphere. The fornix is very soft. The pineal gland contains very little sabulous matter. There are filamentous adhesions between proximate surfaces in the fourth ventricle, and at the base of the brain. One ounce of serum in the ventricles and at the base. Cerebellum thought to be somewhat softened. Its investing arachnoid apparently normal.

Considering the protracted course of the next case, and the comparatively extreme degree of the paralysis of the voluntary muscles, it is remarkable that the functions of the digestive organs were so little impaired, and that the patient was exempted from those sloughing ulcerations which are one of the most striking characteristics of the disease in its severer forms.

Case II.—Mr. —— was a native of the State of New York. He was tall in stature, his hair black, eyes blue, temperament sanguine-bilious, the bilious greatly predominating, constitution *mediocre*. His intellectual faculties were fair, and he received a good English education. Being devoted to mercantile pursuits, he emigrated to a southern State at the age of between twenty-five and thirty years, established himself in business, and was sufficiently successful. He was never married. It was said that his habits were correct, but by persons who had no intimate knowledge of his course of life. His mother was eccentric, but it was asserted that he inherited no predisposition to mental disorder. At the age of thirty-five years he had scarlatina; and at the age of forty-three, what is described as a "slight attack" of paralysis. He lost his property, and became excited with political affairs; but whether prior or subsequently to the commencement of insanity, could not be accurately ascertained.

Having become insane, he was brought by sea to New York. On board the vessel he was so violent that he was most of the time kept in a strait-jacket.

On the 18th of March, 1848, at the age of forty-five years, he was taken as a patient to the Bloomingdale Asylum. He was then emaciated; his skin sallow; the tongue furred and pasty; bowels costive; pupils unequal, the left being the larger; speech imperfect and hesitating; gait faltering. He appeared bewildered; thought he was in Savannah; said he saw an angel on the previous night; would begin to speak, and, forgetting the idea, run to another subject.

He slept but little, at night, during the first few weeks after admission; but he could not bear opiates. One morning his forehead was severely bruised, probably, as has occurred in other cases of the kind, by running against the walls. On being asked how it was done, he said, "The raft slid into the river and many people were killed, but the ladies walked across the plank of the steamboat and were saved." On the 14th of April he said that he was in a southern city, and that on the previous night they "stuck him into a rotunda to sleep." A copy of a New York newspaper being handed to him, he appeared much astonished, and remarked that "it must have come by telegraph." General sensation was then very obtuse. On the 16th, he said that in the night he saw five or six hundred little soldiers, beautifully dressed, and on horseback; they were not larger than his fore-finger, but they "fought the Bostonians courageously, like tigers." His bed being wet, and emitting a strong odour of urine, he was asked the cause of it, and answered that some person opened his window, and a shower coming up, it rained upon him—but it was warm rain. The night was clear. On the 20th, his appetite was good, and he was gaining flesh and improving in general health. He said he had some barrels of the best wine in the world; and, assuming a very earnest, business-like manner, requested to be let out into Broadway, as he was going to the banks, and was afraid he should be too late.

In the summer he took Lugol's solution of iodine; and a seton, which was introduced on the 4th of April, caused a free discharge. He gained flesh, and his general health was good. His mental condition varied, but was at no time much, if any, better than at the time of his admission. He had but little memory of recent events. Soon after a visit from his mother, he said it was more than a year since he had seen her. In the early part of August it was perceived that he had lost the sense of taste. He ate all kinds of food with equal relish. In the early part of September, his feet were œdematous for a few days.

On the 17th of November his pulse was 76, regular; pupils unequal, the left being the larger; appetite voracious; face and feet œdematous; gait unstable. He walked with his feet far apart, like an infant; the grip of the hand and the strength of the arm were feeble; speech considerably impeded, but less so than at some former times. At this time he occasionally tore his bedclothes, and upset the furniture in the room. On the night of the 29th of November, he thought the earth was sinking, and, in order to save himself, he turned his bedstead up upon the side, and seated himself astride it. He said he was thus enabled, by using his utmost exertions, to save himself from being engulfed. His speech was now much more impaired than at any previous time. General sensation was nearly null, but existed to a greater extent upon the legs than upon the superior portions of the body. His feet and hands were somewhat œdematous. He asserted that he could run twenty-five miles in an hour, or walk twenty miles, and that he owned six hundred acres of land at the South and one hundred acres in Harlem, occupying the latter as a barber's shop. Being requested to write a letter to his mother, he sat down, and, after much labour, hesitation, and alteration of orthography, produced a document, of which the following is a copy :—

"Mrs. Deear Motherr
Vder as —— this 29th Jurly
b — o gond to
$18. S. DOOCCKET."

The signature bears no resemblance to the name of the patient, except that the initial letters of the former are the first two of the three which belong to the latter.

There was no material change in his general condition at the time I left the asylum, in May, 1849. Neither was there, as I am informed by my successor, Dr. Nichols, throughout that year. During the whole of his residence in the asylum he never recognised, as an acquaintance, any person except his mother. During the last six months of his life he did not know even her. In the early part of 1850, the power of the voluntary muscles visibly diminished, but most rapidly in the lower extremities. For six months before his death he could not walk without aid. His digestive functions remained but slightly impaired until the 5th of August, 1850, when he was attacked with diarrhœa, and died on the following day. No autopsy.

The third case is exceptional, so far as my observation is concerned, in the striking similarity of its earlier symptoms to those of *mania à potû*. The disease was rapid in its course, and all its other characteristics would probably have soon assumed their worst form had not the patient been carried off in an attack of cerebral congestion.

Case III.—C. —— was a native of Ireland. His constitution was strong, frame robust, stature medium, hair sandy, eyes grey, temperament sanguine, intellect *mediocre*, education common. At the age of about twenty-two he emigrated to America, settled in the city of New York, and established himself as a retailer of liquors. He was subsequently married. He afterwards became addicted to the daily use of alcoholic drinks, though not frequently to intoxication.

In September, 1846, when he was at the age of twenty-eight years, he lost a favourite child, and his friends say that his insanity appeared immediately afterwards. He was subjected to no medical treatment. For four weeks he gradually grew worse: was restless and talkative, and indulged in extravagant schemes of business, made imprudent purchases, and wandered about the city, apparently without any definite object. At length, having determined to go to Ireland, he went to a wharf, jumped into a boat, and rowed himself out into the river. His determination then changing, he leaped into the water, and swam to the shore.

A day or two after this occurrence, and on the 16th of October, he was brought to Bloomingdale Asylum. His friends asserted that he inherited no predisposition to mental disorder, and had always enjoyed good bodily health.

During the first three days after his admission, he had all the symptoms of a person labouring under a severe attack of delirium tremens.

He was excited, sleepless, turbulent; had hallucinations of vision, and would keep no clothing upon himself, excepting a blanket thrown over his head, or wrapped about his body. His tongue was tremulous, his pulse rapid.

After catharsis with cal. et jal., followed by compound cathartic pills, he took mass. ex hydrarg. gr. ij. t. d. and, subsequently, a portion of pulvis purgans. On the 23rd he was so much improved that he was permitted to be in the hall, and to go out of doors; and, on the 24th, he began to take a tonic vegetable infusion. He rapidly gained strength, and on the 29th the medicine was stopped. During this period there was a partial bewilderment in his aspect and manner. He was careless of his personal appearance, at times tore his clothing, and was otherwise mischievous. His appetite was now good. He generally ate voraciously, and required occasional purgatives. This was the only medical treatment to which he was subjected, with the exception that, a few days before his discharge, he took Fowler's solution gtt. v. t. d.

*November* 10*th*. For several days past he has uttered the most extravagant ideas. He now says he owns the Asylum premises, and is worth two hundred thousand billions of dollars. He also declares that he is the head of the Church throughout the world, and is going to turn the earth into a paradise, and manage it all himself.

11*th*. He went to the school-room and wrote a letter to his wife, from which the following extracts are made:—

"I am at the reading school and am one hundred times as smat as any of them they they are the greatest dunces in Eternity I shall commence travelling next week Please God and the first place I will go to is to my native own green Isle." . . . . . "I would not trust the word no but the Oath of G. and O. I wd not Trust them in an Empty room or a room full of Mill stones I am tak as many friends as go with me By their Paying Expences it wud not not mak much of a difference I shl have High life all over the continet and all the Corners in the World which I will make a Parridise of all the world and Have shepherds to take care of them so that has Plenty

Resp ful

C.—— ——

Head of the C. Church all over the world

C.—— ——."

12*th*. The pupils are unequal, the right being the larger. There is an evident stammering in his speech, and general sensation is so obtuse that he can barely feel the most severe pinch.

He says he is worth ten times as much as John Jacob Astor. Being seen to make some strange gesticulations, he was asked what he was doing, and answered that he was blowing himself up; that he could blow himself so large that he would be thirty feet in height, or reduce himself to the size of twopence. On being requested to blow himself up, he put the end of the forefinger of each hand into the ear of the corresponding side, elevated his head, rolled his eyeballs as far upwards as possible, compressed his mouth, puffed up his cheeks with air, stretched himself upwards, standing upon tiptoe, and thus exerted himself until his body was in a general tremor. Upon being told that that was enough, he said, "Oh, that is nothing; I only went up to nine feet."

16*th.* He is endeavouring, with but little success, to sing; says he hears and sees music throughout his body, and can sing better than any man at the Italian opera. He asked for writing materials, for the purpose of "corresponding with all the different Governments on the subject of converting the world into a paradise." Being permitted in the afternoon to go to the school, he wrote a long letter to his wife. It was so badly written as to be almost illegible, and closed with a postscript consisting of two verses of pretended poetry, but so far as it could be deciphered, contained no rhyme and but little reason. From this time his delusions continued unchanged.

23*rd.* Sphincter of the bladder apparently paralysed. He says he can swell to the height of more than a hundred feet. He is very noisy at night; chews and swallows pieces of woollen rags, picks his clothes to pieces in the daytime, and empties the straw from his bed at night.

25*th.* He shuts his eyes, and says he sees "gold and all the brilliants in their shape and lustre manufactured;" says he weighs five hundred pounds, can run thirty miles in an hour, and walk twenty. He frequently "blows himself up;" attempts to sing, talks of his wealth and of his proposed conversion of the world into a paradise. He exhibits little or no interest in his relatives and friends.

On the 26th he fell into a state of coma, with very slight spasms of the limbs of the right side. This resisted the usual remedies for more than twelve hours, when he partially revived. He continued in bed, rarely speaking, and with but imperfect use of the right arm and leg, until the 29th, when he was removed from the asylum, and died at home on the following day.

No autopsy.

The subjoined is the most remarkable case of the kind that has ever fallen under my observation. It is the only case of recovery from the partio-general paralysis that I have ever known, and the second of which I have ever heard as occurring in this country. Mr. Calmeil, who first minutely described the disease, and who had for more than twenty years been connected with the Hospital for the Insane at Charenton, near Paris, where hundreds, perhaps thousands, of cases had been treated by him, informed me, in 1849, that he had never known a case of complete recovery. He had had patients who improved sufficiently to return to their homes, and, in some instances, to pursue their occupations, but in every one of them the disease had resumed its course.

Case IV.—Mr. —— was a native and resident of one of the interior counties of the State of New York. He was of medium stature, with brown hair, grey eyes, and lymphatico-nervo-sanguine temperament. His constitution was strong; his intellect above mediocrity. After pursuing a classical course of study, he read and practised law, and became eminent in his profession. He was married at the age of thirty-four years. Although not intemperate, according to the common acceptation of the term, yet it was said that he "liked good living, and indulged freely in the luxuries of the table." One of his paternal uncles was insane, and a maternal aunt was affected with melancholia.

In August, 1847, he was much afflicted by the death of a favourite child; and in September, having involved himself in pecuniary difficulties, he became melancholy. In the early part of 1848 he had an epileptiform fit, which was followed by another upon the same day, and, subsequently, by several others. It was said, however, by his friends, that previously to this his speech had become defective, and the muscles of his arms so much impaired in their action that he was unable to write. His disease continued gradually but slowly to progress, and for some time he was under the care of the local physicians. On the 30th of July, 1848, at the age of forty-two years, he was received into the Bloomingdale Asylum.

At the time of admission he was much excited, constantly in motion, walking to and fro, talking incessantly and incoherently, mostly upon pecuniary matters. He wanted to go to Wall-street, where he said he would purchase $35,000 worth of railroad stock, and make a great speculation. He spoke rapidly, but frequently dropped a syllable, and sometimes hesitated, from inability to utter a word. The pupils were contracted, but of equal size; tongue furred; pulse somewhat accelerated. After the administration of a dose of pulvis purgans, he was put upon the use of twenty drops of antimonial wine, with ten drops of the tincture of digitalis, three times daily.

31*st*. He is still much excited, shouting that he wishes to get out of the house and go to Wall-street. His speech is more imperfect than it was yesterday. No evacuation of the bowels. ℞.—Cal. et jal. āā grs. x.

*August* 1*st*. There having been but a slight alvine movement, another portion of pulvis purgans was administered. This produced free catharsis, and his excitement was considerably subdued.

6*th*. The pupil of his left eye is larger than that of the right, and there is an evident partial paralysis of all his limbs.

11*th*. His excitement has almost entirely subsided, and the paralysis has so far increased that he cannot walk without support. Stop the vin. ant. and tinct. digital., and give a tonic vegetable infusion three times daily.

14*th*. His ideas of wealth, of station, and of power have been constantly increasing since his admission. He now says that he began business with a borrowed capital of three hundred dollars, and from that has accumulated a fortune of five millions; that in the town of Oswego he has one hundred and fifty mills, each containing five runs of stone, and the whole turning out twenty-five thousand barrels of flour each week; that a million of dollars has been cleared by this operation; that he has seven ships at sea—four of them on whaling voyages, two bound to China for cargoes of tea, and one to the Mediterranean for fruit; that he has purchased the whole of the United States, except New York and Philadelphia, together with the wheat lands in Canada, and the whole of Mexico, for all of which he paid but one million of dollars; that he owns two coal-mines, one in Virginia and the other in Mexico, all the copper-mines in Wisconsin, one gold-mine in Africa, all of those in Mexico, as well as all other mines of gold and of iron, and that his income from each of these mines is seventy thousand dollars in three weeks; that among the rest of his property are—1, the Bank of Milwaukie, with a capital of three hundred thousand dollars; 2, three hundred thousand dollars invested at twelve per cent. interest in New York; 3, stock to the value of five and a half millions in the Hudson River Railroad; 4, a factory in one of the towns upon the Hudson River; and that he is about to establish a bank in New York, with a capital of two millions of dollars.

He asserts that he is a Judge of the Supreme Court of the State of New York, and a member elect of the next Congress; that he is to be appointed minister to England; and that he shall be elected as the next Governor of the State, and the next President of the United States after General Taylor. He proposes to start, to-morrow, on a tour to the Catskill Mountain House, the Thousand Islands, Quebec, Montreal, Oswego, Falls of Niagara, Ohio, Washington, Florida, Mexico, and Buenos Ayres, returning by the way of Mexico, Mississippi, Illinois, and Oregon. This journey, he thinks, will occupy his time for four weeks. He intends, after it is completed, to start for Europe, and spend two years in England, two in France, one in Switzerland, one in Germany, one in Sweden, three in Russia, one in Norway, one in Turkey — in Constantinople —("Con-con-stan-no-nople," as his impaired enunciation makes it), and one week in Africa, making, in all, eighteen years. He proposes to take his wife and children to Russia with him, in a steamer of one thousand tons burthen, which he will have built expressly for the purpose, and named for himself and wife. He will freight it homeward with English goods, which will yield a profit of $100,000. On its second voyage, he intends to return and to build twenty houses, at a cost of $10,000 each, on one of the docks in New York.

17*th*. His general sensation is obtuse; his taste imperfect. A portion of the sulphate of magnesia being prescribed for him, it was made into a strong solution, which he drank, saying that it was "first-rate Congress water."

21*st*. The paralysis has extended to the sphincters of the bladder and rectum. The patient's speech is variable, being much more imperfect upon some days than upon others. His memory of recent events is almost entirely destroyed. He says that he has invited several guests, among whom are God and Van Buren, to dinner; and that one of his whaling vessels arrived yesterday with twelve hundred barrels of oil, upon which he will make a nett profit of fifty thousands of dollars. On being informed of the recent destructive fire in Albany, he remarked that he did not "own any of the buildings that were burned, except the Eagle Hotel, the Mansion House, the Townsend House, and the Odeon, which are all insured for their full value." He added, that he has "bought all the land of the burned district, and is going to build it up with marble;" and that he will "immediately give one hundred dollars to the sufferers, and fifty thousand dollars by-and-bye."

There are many sores upon different parts of his body, some of them apparently having arisen without any external cause, and others the ulceration of places upon which the skin was abraded in the course of his period of high excitement. Attempting to write his name, his hand is unsteady, moving by partial jerks; and although one or two of the letters are pretty accurately made, others are very imperfect, several are entirely omitted, and there are some unmeaning marks. It takes him probably five times as long to write it as it did prior to this disease. On the second attempt he is somewhat more successful, but his writing is no better than that of a child in his first essay upon a connected fine-hand copy.

*September* 1. The left pupil is larger than the right; but both are contracted.

3*rd*. He says he is worth ten millions of dollars; that the Lord came down to him; that he is now sixty-five years old, but the Lord will make him only twenty-five.

5*th*. Besides his fanciful ideas of wealth, he now has many religious delusions. He often calls himself a bishop, or a clergyman, and asserts that he is going to preach in Trinity Church.

17*th*. He says that God is up in the room, on his throne, and is going to preach to-day; asks us to go up and see him. A seton was this day inserted in the back of the neck.

18*th*. He talked as follows: "I went up to God one day, and said, 'God, what is the reason that —— and —— are in hell?' His answer was, 'They are not in the right line of succession with the church.' 'Well,' says I, 'what does the devil do with them when they are first put in there?' He said they were first ground down with fire and red-hot iron; afterwards they were ground down with spirits of turpentine and saltpetre. Don't you think that will make them smart?" He then proceeded to give an account of his wealth, and concluded by saying that he was the most eloquent lawyer in the world.

27*th*. His pulse is always rapid. It is now 124 per minute, small and regular; pupils nearly equal, tongue slightly coated, bowels regular, the sphincters under voluntary control, general sensation less obtuse than it has been. He writes better than he did, and can stand alone, but cannot walk without assistance. Being asked how much he was worth, he answered, "Nine hundred thousand dollars," hesitated a moment, and then added, "No; God says it is ten millions. I have made ten thousand four hundred dollars while you have been sitting there; and I own a million dollars' worth of jewels." He then said that he goes up to Heaven to see his father, and offered to take his mother up with him.

The seton produced a considerable discharge throughout the month. The tonic infusion was stopped in the early part of October, and followed, through a large part of the month, by alterative doses of the bichloride of mercury. Under this treatment the discharge from the seton almost entirely ceased, most of the sores upon the body healed, and the appetite and digestion of the patient continued to be pretty good. In the latter part of the month he was attacked with diarrhœa, which was subdued by opiates. All the characteristic symptoms of the paralytic insanity varied from day to day, but subsequently to the 27th of September the patient was not at any time better than upon that day. The general character of his delusions remained unchanged. At one time he enumerated the different offices of which he imagined himself to be the acting incumbent. Among them were the presidencies of several banks, insurance offices, and railroads; a number of bishoprics; offices under the national government, &c. &c. He made the aggregate salaries $76,000 per annum.

On the 3rd of November, 1848, the patient was removed from the Bloomingdale Asylum to Dr. Macdonald's private institution, at Flushing. There, after a residence of some time, he began to amend, and at the end of a few months was discharged, recovered. Dr. Macdonald died soon afterwards, and I had no opportunity of conversing with him in regard to this very remarkable case. I am informed, however, by Dr. Benjamin Ogden, that no special treatment was pursued which was supposed to have effected a cure, but that Dr. M. attributed the patient's recovery solely to an effort of Nature.

This gentleman is still living. He is in excellent health, both physical and mental, and is engaged in an extensive and successful business.

In the following case, the symptoms, not only in its earlier periods, but along its course, were such as to lead the experienced observer to the prognosis of paralysis; and yet, although the progress of the disease was comparatively slow, and although some of the other most peculiar characteristics of the partio-general paralysis were present, the paralysis itself never appeared:—

Case V.—Mr. ———, a native of the interior of New York, was of medium stature. He had brown hair, grey eyes, and bilious-nervous temperament, the nervous greatly predominating. His talents were fair, and he received a good common education. He was active, intelligent, and of mild disposition, though excitable. His mother once had an attack of insanity. He entered into business, and when quite young accumulated great wealth by speculating in real estate during the years 1835 and 1836. This fortune, however, he subsequently lost; and afterwards engaged in various kinds of business. He was married and had children. In the winter of 1845-46 he came to the city of New York, in the hope of finding employment. Soon after his arrival, his friends perceived that he was eccentric, wilful, and easily excited; more than usually talkative; self-complacent when speaking of his business capacity, and elated with great hopes for the future. These symptoms increased. He began to make imprudent purchases; gave away his money, lost sleep, and grew more and more excited until the 23rd of February, 1846, when, at the age of thirty-six years, he was brought to the Bloomingdale Asylum. His friends stated that he had had a cough ever since the preceding summer.

*State when admitted.*—Emaciated, somewhat sallow; pupils natural, tongue slightly furred, bowels costive, pulse 110. He is restless, and very talkative, but shows no disposition to be violent. He consents to remain, but thinks that "placing a man, so well as he is, in a Lunatic Asylum, is one of the most ridiculous farces ever imagined." His general conversation is quite rational, and no attempt is made to elicit his exalted ideas. Before his friends leave, however, he in great good-humour takes some papers from his hat, and requests the doctor to look at some poetry which he has this day been writing. The paper contains six stanzas, the first three of which he says were written by his favourite author, Mr. Tupper. The others are a parody upon them composed by himself. After reading these lines, and hearing a history of his case, I told his friends that I thought there was but little hope of his recovery.

*February* 24*th.* ℞.—Blue mass. gr. ij. t. d., with an aloetic pill morning and evening.

*March* 1. Skin more natural, and he looks less worn; tongue clean, appetite and digestion good. Stop mass. and pill. ℞.—Tinct. opii gtt. x. t. d.

4*th.* Bears the opiate well; sleeps sufficiently. Increase tinct. opii.

Four days after admission he wrote a letter in reference to some mineral lands to a gentleman in the northern part of the State, with whom he was entirely unacquainted, requesting him to take men and teams to those lands, procure one or two thousand barrels or boxes of all kinds of minerals, and send them to him in New York; stating, furthermore, that if the said gentleman had not money enough to accomplish this object, he might draw upon him. He then proceeds, by way of introducing himself to the stranger to whom this letter is directed, to give a genealogical history of himself and of his wife. He says, that if the minerals should prove to be rich and the lands valuable, the county in which they are situated will become more populous. "We will," says he, "put a bank at your place or in Peru, and it would be a good place for a college for the north of this State, better calculated than any in the State now; for it might be used for the poor of the State, as well as those who could handsomely pay. I speak of this as an inclination, and not anything which would trouble me at all if it should not be worth anything. And as to the sum to be paid to the noble man, the owner of the farm, the soldier of the great Revolution —why, I think I would not feel a sigh to pay him $200 a year as long as he lives, without any interest at all, if it would do him good, for I feel perfectly well off, and it would give me much pleasure and contentment to do such a thing."

About the time of the date of the foregoing communication, after reading the advertisements of several valuable houses that were to let in the city of New York, he wrote to the owners, advising them to furnish the houses, as they would then rent more profitably than if unfurnished, and made some preliminary propositions in regard to hiring them. He subsequently wrote the following letter:—

"New York, *March* 20, 1846.

"To the Hon. Daniel Webster.

"Sir,—As a stranger, and having some business to have done at Washington, which I know to be of great importance to me, if not to our country. . . . For three years I have known what I now write, yet have said nothing; but now, as the great and good men of both parties, conservatives, are all together, I thought it of great importance; and it is this: That by using the bright sands of the sea-coast, and the small, round, clean stones, or other hard matters, with water-lime, you can make a road from here to the upper part of Oregon, in a month, or less; because water-lime, mixed with clean stone or glass, or anything solid, will make a road much better than a railroad. So far in a month, for instance, make it soft and mix it clean, and throw it upon the ground as far as you choose, and make it smooth, and, as soon as it is dry, it is, in my opinion, harder than rock. And should the great men of our great democratic nation, now altogether to do right, believe surely, as I do, that, in one day, I could, with that mixture, by the aid of good builders, make one hundred ships a day. And now, suppose a ship was planned large enough to carry thousands. Make it three feet thick and one hundred feet wide, and flat on the bottom, having large places all along its side or bottom, to take it up if necessary, and put down again. Well, it would require no ballast; and round the sides, from the bottom to the top, and while it is soft, at the bottom fix a keel, as low as profitable, that can at any time be hauled up for other purposes; such a ship, in my opinion, would draw but little. And, as far as war was concerned, no common shot or ball could hurt any one; for it is a rock, smooth, and the balls would slide under. Now, build as many as you please, in a month, and put them together, and in two or three days they could reach England, and everything upon the ocean could be taken without trouble, or anything else. The reason they would, in my opinion, go so fast, is that they could draw no water, laying so flat, with a deep tiller, if it would be thought right, and with engines of the screw to give them their power; when they were wanted for something else, it would be well to have the engine screw put in the bottom, so that you could bring it within the ship, and have rollers under, which would cross any land one hundred feet wide; and make a railroad or road of hard rock, and as fast as the stuff could be thrown out (I mean the sand-lime and stone), the engine within would roll the rollers under the ship, and make the road smooth and ready for use as soon as it was dry. And, before it was dry, the same material would make a fence as high as would be necessary for anything, by sticking them down when wet. Carriages, and everything, almost, could be made, and will be, and buildings (safe from all fire) which now cost so much, could be built by my patent for a little. Now, in my opinion, should it be thought right, and above all question, in my opinion England could be made a state of this Union, and all Europe, and this hemisphere, and the whole world, could easily be made one democratic kingdom. And now it is useless for me to say more at present. I have wished to be secured in the Patent Office for this matter for all time. All I have acted upon was a trial in digging a hole for a post, and putting it in, and throwing in this material, and it became stone. If such an arrangement could be made with our great men, say H. C. C., the Secretary of State, Mr. A., and the best in Washington, why, I think, without spilling any blood, an arrangement might be made with England, letting them have their titles they now have, and making them and their great men only as farces, our own great men to rule the world. I believe it was Napoleon who said, before thirty years, that Europe would be democratic or Russian. Now, I have been reading the great argument of Senator C. upon our position with Great Britain—wonderfully correct, and, with one exception, true. But he thinks to possess Mexico. It would cost millions. Why, it is all wrong, for it would cost nothing to speak about. If it would be allowed by our Union for a man to undertake the control of Mexico on his own account, I am sure it could be done in a month, and could be done without asking a cent from the country. I would begin a road with my mixture at Washington, *via* New Orleans, and, at the same time, make arrangements with the wire telegraph to use it under ground instead of above, for the use of the Government. There would be no hindrance from water or land in running such a road through to Mexico, with such a fence that few could get over on each side of it, and no guns could hurt or shatter the machine or ships. No blood would be spilt, but all taken.

"Now, not to let it be known that ships and other things are made in this way, it would be extremely necessary that the patent should be concealed, and the ships covered with sheet iron, and call them iron ships. And as to Canada and New Brunswick, it would be all the same, and I truly believe, if the question was placed by the great men of this country at England, with our ships in sight, that they would be satisfied to become part of our Government, and in doing this without much trouble. It would insure unto the United States the government of the whole world, making it democratic, or allowing the great men of their country to join with ours in the government; and it would be a wonderful affair in respect to the religion of our Maker, for now the news of the arrival from Europe is, that England is now at war in the East, and many thousands have been killed lately; and now is the time to put a stop to this business. If I am right in my idea of the great and wonderful power our Maker has given to this country, no argument, in my opinion, can be made which can be a conviction of truth against this: that the United States should do her most to gain the control of all they can, simply for the defence of their own liberty, and the liberty of the whole world.

"I shall say no more at present, but, at all events, as soon as you get this patented for me, and if you think I am wrong in my ideas of *right*, why keep this a secret, and return it to me. I would have no man see it, if your opinion is against it, as far as the Goverment is concerned.

"Yours, respectfully, —— —— ——.

"P.S.—Show this to Calhoun, and let me hear from you immediately."

He wrote several letters to his wife. The following extracts are made from one of them:—

"My happiest moment in life is now, I am well beyond all question, and healthyer than I ever was before.

"I am so well that I have grown so strong and healthy that you would hardly know me. I was measured yesterday and found myself at least six feet high with boots on, my whole body looks as straight as it could be, and I cannot alter it. I feel great in my power which has been given to me by my Maker, for there is nothing I can not do in business and the following year will test the question. . . . . I can write any thing, poetry, argument, and can sing as well as I wish, and sing without knowing any thing, but with my ear, when I get through this I will give you a happy song, of three or four verses which I think will be suitable to the occasion. I have written to W—— upon country matters. . . . . . I can follow Tupper and I think I can do what he has done. . . . . .

"And now to thro' off all nonsense I will write a few verses as I said I would

"Dear blessed sweet —— a dear Queen
Always so beautiful, as the sun shining
Upon the Earth which our Maker, green
Has given to you and to me, rising.
Upon this wonderful world beautifully seen

With our eyes beautifully shining, devising
Our word of the great truth, upon which we lean
Given by the Lamb of our Maker so, rising
Above the great world, by our Redeemer's will,
That you and me, with holy thoughts, sighing
Away our delightful selves, so still
To our Redeemers; wonderful rising
From death, to his everlasting good
Which wakes you dearest, and your loving ——.
In this beautiful world our hearts always good,
To Our Redeemer, which always will make us
Nature Nobleman, and quean with our
Dear blessed hearts in one hand, in one hand."

*May 29th.* He has gained much flesh; his appetite, digestion, and general health are very good, and there appears to be no indication for further medical treatment.

From the time of his admission his restlessness and excitement have gradually subsided. He is perfectly calm; and a stranger, in a short conversation with him, might not perceive anything peculiar. To those around him, however, he frequently enlarges upon his magnificent schemes. He imagines that he has more talent and skill in everything than any other man. In literature, particularly, he believes no one to be his equal. He really plays skilfully at cards and nine-pins, but is irritated at the least opposition.

After this he continued very slowly to improve, although he was subjected to no further medical treatment. He had the liberty of the premises, upon parole, and passed much of his time, during the summer, sitting or lying in the shade, reading. He less and less frequently alluded to his extravagant notions, and throughout most of the winter could not be induced either to speak or write anything in reference to them. It was believed, however, that he still secretly entertained some of them; and a degree of self-complacency was still exhibited. In the course of the winter he did considerable writing for the officers of the institution, copying documents in a good, legible, and firm hand.

Discharged, much improved, January 2, 1847. He went to his home. About two months afterwards he called at the asylum, and appeared to be in nearly the same condition as when he was discharged. He now attempted to obtain employment in the city, but his friends were obliged to send him again to the country, as he was considered unfit for business. On the 20th of May, 1847, he was taken, hand-cuffed, to the Utica Asylum. For a time he was excited and somewhat destructive. His ideas were exalted, and in the daytime he was almost constantly in motion. He said he was going to be President of the United States; that he owned the State of New York, and was going to plough it all with a plough made of cement. He pretended to communicate with his wife and with the Government, by telegraphic dispatches. He thought his food was poisoned, and, at length, refused to eat, so that it became necessary to feed him. There was no evident defect in his speech or gait. In the autumn he became more calm, and joined others in playing cards; but even in his best condition, if he was alone, he was constantly walking to and fro, rubbing his hands, and pretending to be making worlds.

After a few weeks he became more excited, and it was necessary to confine him in a darkened room, and, at length, to his bed. Here, during the day, he still talked almost incessantly—the making of worlds being a prevailing topic. In the winter he had an attack of cerebral congestion, unaccompanied by spasms. He roused from the immediate effects of this, but his mind was much more impaired than before. Afterwards he had illusions and delusions simulating those of delirium tremens. He imagined that he saw devils, and struggled in encounters with them.

During the last few weeks of his life it became necessary to feed him, and his bowels were moved only under the effect of powerful cathartics. He was emaciated and ghastly, and his mental faculties almost entirely prostrate. He died on May 2nd, 1848.

The principal pathological appearances of the brain were as follows :—Thickening and opacity of the arachnoid pretty general; blood-vessels enlarged; pia mater much injected; about four ounces of serum in the cranial cavity; substance of the brain generally softened.

In the autumn of 1848, I was requested by Dr. H. D. Bulkley to see a patient, then under his medical care at the New York Hospital, some of the symptoms of whose case were very similar to those of the partio-general paralysis. The man died soon afterwards, and Dr. J. B. Arden, formerly one of the house-physicians of the hospital, furnished me with the following brief history of the case :—

D—— ——, æt. thirty-three years, resident of New York, boatman. About six months ago the patient had a slight apoplectic attack, from which he so far recovered as to be able to walk about in three or four weeks; but he has never completely recovered the faculties of his mind. He has lost his memory, and the ability to recall the appropriate names of objects. He has not complete control over his lower extremities; walks with difficulty and unsteadiness; does not complain of pain in the head. The pupil of the left eye is much the more dilated, but is slightly acted upon by light. General health good.

*Nov. 2nd.* Patient remains about the same; has no pains; walks about the hall with the aid of a stick.

*Dec. 4th.* Patient last night had an apoplectic attack, with tonic convulsions, and in about six hours died.

*Autopsy, eighteen hours after death.*—On opening the cavity of the cranium, there was found a large effusion of blood under the arachnoid membrane, and around the medulla oblongata. The lateral and fourth ventricles were filled with fluid blood, in which were some coagula. There was no marked softening of the brain. The right vertebral and the basilar arteries presented an appearance resembling a varicose vein, or like a string of beads; in other words, there was aneurism of these vessels. The basilar artery was, in one point, as large as a pea, and this enlargement was situated under the pons Varolii. Other organs healthy, as far as examined.

